# Altered Functional Activation Is Associated With Speech Dysfunction in People With Multiple Sclerosis

**DOI:** 10.1162/NOL.a.23

**Published:** 2025-11-13

**Authors:** Katherine Hope Kenyon, Frederique Boonstra, Gustavo Noffs, Angela Morgan, Adam Vogel, Scott Kolbe, Anneke van der Walt

**Affiliations:** Monash University, Melbourne, Victoria, Australia; Murdoch Children’s Research Institute, Melbourne, Victoria, Australia; University of Melbourne, Melbourne, Victoria, Australia; Royal Melbourne Institute of Technology, Melbourne, Victoria, Australia

**Keywords:** dysarthria, multiple sclerosis, neuroimaging, neurolinguistics, speech production

## Abstract

Dysarthria is a motor speech disorder that is a common symptom of cerebellar dysfunction in people with multiple sclerosis (pwMS). Despite its prevalence, little is known regarding changes in brain functioning associated with dysarthria in this cohort. Management strategies for cerebellar symptoms such as dysarthria are also limited. Fifty-five pwMS and 14 healthy controls participated in this study. We used fMRI to assess changes in speech related functional activation associated with MS, and split our MS cohort into people with and without dysarthria, and with and without cerebellar dysfunction clinically evident as upper limb action tremor. We found that pwMS performed worse on speech production tasks and had overall lower functional activation while preparing for speech than controls. Furthermore, pwMS require additional recruitment of the left Brodmann areas 45 and 46, key motor speech regions, during speech production compared to healthy controls. MS participants presenting with both dysarthria and action tremor performed worst on speech production tasks. These participants had lower functional activation during speech production compared to other MS participants. People with multiple sclerosis display altered functional activation of motor speech areas during speech production, either due to MS injury or reduced activity during preparation. Compensatory activation is reduced in those with both clinical dysarthria and action tremor compared to MS controls and those with tremor only, likely due to more advanced MS.

## INTRODUCTION

Multiple sclerosis (MS) is an autoimmune neurodegenerative disease of the brain and spinal cord (Wilkins, 2017). MS attacks the myelinated axons within the central nervous system, causing the inhibition of transmitted neural signals (Chiaravalloti & DeLuca, 2008). Genetic, geographical, environmental and socioeconomic factors all influence a person’s risk of developing MS, alongside lifestyle factors such as smoking, obesity and Epstein-Barr virus infection (Dobson & Giovannoni, 2019). For the vast majority of people with MS (pwMS), symptoms first present as attacks or relapses, thus receiving a diagnosis of relapsing-remitting MS (RRMS). Approximately 80% of people diagnosed with RRMS end up experiencing a constant progression of symptoms within 25 years, known as secondary progressive MS (Brassington & Marsh, 1998; Gelfand, 2014). Symptoms of MS are dependent on the regions damaged, and thus the disease is highly heterogeneous. However, particular regions of the brain are commonly impacted by the demyelination, inflammatory and neurodegenerative processes of MS. Over 80% of pwMS experience cerebellar damage (Le et al., 2021), impacting sensory, motor, cognitive and behavioural processes (Parmar et al., 2018). Cerebellar symptoms such as ataxia, dysarthria (motor speech disorder) and action tremor are associated with increased disability and worse prognosis (Wilkins, 2017). Dysarthria and tremor are closely associated in the MS population (Boonstra et al., 2017). Both symptoms are seen in up to half of all pwMS and occur most frequently in people with secondary progressive disease (Boonstra, Noffs, et al., 2020; Noffs et al., 2018; Piacentini et al., 2014; Smyrni et al., 2025). Relapses of cerebellar symptoms are associated with greater risk of disability accrual, but treatment and management options for symptoms such as tremor and dysarthria are limited (Wilkins, 2017). Dysarthria is a speech disorder resulting from orofacial muscle weakness and incoordination caused by lesions in motor regions of the cortex and cerebellum (Hartelius et al., 2000). It is characterized by slowed speech, abnormal temporal patterning, scanning speech and articulation difficulties (Hartelius et al., 1997; Noffs et al., 2021). Dysarthria can impact one’s confidence and ability to engage fully in social situations (Yorkston et al., 2014), which can in turn negatively influence self-image and quality of life (Enderby, 2013; Noffs et al., 2021; Piacentini et al., 2014). Despite the impact that dysarthria has on pwMS, speech dysfunction in this population is still not well understood, especially in terms of neurological involvement (Rusz et al., 2018). Research into speech in MS has historically been limited to perceptual tests (Sonkaya & Bayazit, 2018), but recent studies have applied neuroimaging techniques to investigate the pathophysiology of dysarthria in pwMS (Noffs et al., 2021).

Altered functional activity and connectivity measured using functional magnetic resonance imaging (fMRI) are characteristic of MS (Mormina et al., 2017). fMRI can be used to identify functional abnormalities in the brain in pwMS and can track how these abnormalities vary over the course of the disease (Filippi & Rocca, 2013). Boonstra, Noffs, et al. (2020) reported pwMS with upper limb action tremor presented with increased activation in the premotor cortex and supplementary motor area (SMA) during an upper limb motor task compared to pwMS without tremor. In contrast, reduced functional activation during a visually guided motor task was seen in the superior parietal lobule, inferior temporal gyrus, inferior occipital gyrus and cerebellum in pwMS (Strik et al., 2021). That study also established that motor-related functional variation varies with MS disease severity, as measured through the Expanded Disability Status Scale (EDSS; Strik et al., 2021).

In addition to the EDSS, the Kurtzke functional system scores are used to address different areas of neurological dysfunction (Kurtzke, 1983). For symptoms such as tremor and dysarthria, cerebellar and brainstem functional system scores can be used to gauge the level of disability or dysfunction in pwMS (Boonstra, Evans, et al., 2020; Kenyon et al., 2023; Noffs et al., 2021). Similarly, the scale for the Assessment And Rating of Ataxia (SARA) is used to assess cerebellar dysfunction and evaluates symptoms such as speech disturbance, tremor, balance and gait changes in neurological conditions such as MS (Kenyon et al., 2023; Noffs et al., 2020; Salcı et al., 2017; Subramony, 2007).

There is limited research into changes in brain activation during speech, or more specifically dysarthria, in pwMS. fMRI has, however, been used to examine the relationship between activation and dysarthria in other neurological conditions. Increased activation during speech is seen in people with dysarthria due to childhood brain injury compared with people with childhood brain injury without dysarthria (Morgan et al., 2013). This increase in activation, specifically in the left inferior frontal gyrus or left Brodmann area 45 (BA45), correlated with speech outcome in the dysarthric group. Further, there are diverse findings regarding functional activity during speech in people with dysarthria due to Parkinson’s disease. During a passage reading task, people with Parkinson’s and dysarthria showed decreased dorsal premotor and motor cortical activation (Narayana et al., 2020). Increased functional activity in the orofacial sensorimotor cortices is observed in the same population (Rektorova et al., 2007). These varying results illustrate the lack of consensus regarding the direction of change in activation associated with dysarthria.

The present study aimed to determine the pathophysiology of speech in MS and to assess the role of cerebellar dysfunction in dysarthria in MS. To do this, we compared functional activation of the cerebellum and connected motor speech regions of the cerebrum in pwMS with that of healthy controls (HC). We also compared the level and region of activation during speech in pwMS both with and without clinical dysarthria, and with and without upper limb action tremor (considered a marker for cerebellar dysfunction; Boonstra, Noffs, et al., 2020; Boonstra et al., 2018; Koch et al., 2007).

## MATERIALS AND METHODS

### Participants

Sixty-three pwMS initially underwent MRI scanning. However, speech data from the fMRI word repetition task was lost for eight MS participants due to technical difficulties, with a further four missing data from one run. Thus, a total of 55 pwMS and 14 HC aged 18–65 years old were included in the study. All participants were native English speakers. Of the MS participants, nine presented with clinical signs of dysarthria, indicated by a speech naturalness score ≥1. Eighteen MS participants presented with upper limb action tremor, and 10 presented with both dysarthria and tremor. The remaining 18 MS participants acted as an MS control group with no clinical signs of dysarthria or action tremor. Participants with and without tremor were age- and sex-matched. See Table 2 in the Results for a detailed breakdown of participant groups.

### Clinical Assessment

Each participant underwent several assessments, including taking of general medical history, EDSS examination to assess level of disability. The associated cerebellar and brainstem functional system score (CBFSS) was calculated. Cerebellar functioning was measured using the SARA (Kenyon et al., 2023; Salcı et al., 2017). HC scored zero on SARA, EDSS and CBFSS assessment, indicating no impairment. For MS participants with action tremor, tremor severity was rated using the Bain score (Bain et al., 1993). Brief descriptions of each clinical measure can be found in Table 1.

**Table 1.** Clinical and speech metrics included in analysis

Item	Type	Measures	Dysfunction indicated by
EDSS	Clinical	Multiple sclerosis disease severity	Higher score
CBFSS	Clinical	Cerebellar and brainstem dysfunction in multiple sclerosis	Higher score
SARA	Clinical	Cerebellar dysfunction and ataxia	Higher score
Bain	Clinical	Tremor severity	Higher score
DDK rate	Acoustic	Number of syllables spoken per second	Lower score
Read rate	Acoustic	Speed of reading a short paragraph aloud	Lower score
Naturalness	Perceptual	How typical the participant’s speech sounds, measure of dysarthria	Higher score
Vowel *f*0 CoV	Acoustic	Frequency variability, voice control	Higher score
Prolonged intervals	Perceptual	Total pause time/task time	Higher score
SARA speech	Perceptual	SARA subscore assessing cerebellar speech dysfunction	Higher score
Composite EDSS	Acoustic	Speech-related neurological dysfunction (Noffs et al., 2021)	Higher score
Composite SARA	Acoustic	Speech-related cerebellar dysfunction (Noffs et al., 2020)	Higher score

*Note*. EDSS = Expanded Disability Status Scale; CBFSS = Cerebellar and Brainstem Functional System Score; SARA = Scale for the Assessment and Rating of Ataxia; DDK = diadochokinetic; *f*0 CoV = fundamental frequency coefficient of variation.

### Speech Assessment and Imaging Task

#### Selection of speech metrics

Speech metrics were chosen based on previous literature (Kenyon, Strik, et al., 2024; Noffs et al., 2020, 2021; Rusz et al., 2018). Functional speech assessments included the acoustic metrics of number of syllables pronounced per second during a speech diadochokinetic task (DDK rate), reading rate of a short passage (read rate), and vowel fundamental frequency instability during sustained phonation (vowel *f*0 CoV). Two composite speech measures associated with cerebellar dysfunction and disease severity were also included. These were created by identifying speech measures predictive of MS disease severity (composite EDSS) or cerebellar dysfunction (composite SARA) and calculating a composite score through forward stepwise linear regression (Noffs et al., 2020, 2021). We additionally isolated the speech subscore from the SARA as a perceptual measure of cerebellar speech dysfunction. Participants were also perceptually scored by a clinician on prolonged speech intervals during an unscripted one-minute monologue and given a speech naturalness score between zero (*normal*) and four (*severely disordered*). A score over one was taken as indicative of dysarthria (Noffs et al., 2021). Descriptions of each speech measure can be found in Table 1. For a detailed description of acoustic and perceptual speech analysis, see Noffs et al. (2021) and Noffs et al. (2020).

#### Imaging speech task

**Figure 1.**
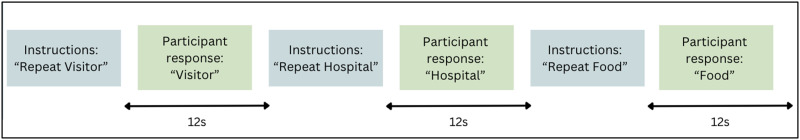
Event-related block design for speech task. Word order is pseudorandomised (Kenyon, Boonstra, et al., 2024). Each instruction is given 12 s apart. The prepare condition is shown in blue, and the speech condition is shown in green.

Imaging data were collected while participants completed a 6-minute speech protocol. Participants listened to an audio recording that presented two runs of 30 single words, preceded by the instruction to either “listen” or “repeat” the word (Morgan et al., 2013; see Figure 1 and Supplementary Table S1 in the Supporting Information, available at https://doi.org/10.1162/NOL.a.23). The words and word order differed between each run, providing a dataset of 60 pseudorandomised words per participant. For this study, we focused only on the “repeat” aspect of the task. The timing of the “repeat” instructions was used to determine timing for brain activation associated with speech preparation. Individual participant response timing was similarly used to determine the timing for brain activation associated with motor speech production.

### MRI Acquisition

At baseline, all participants underwent 3T MRI (TrioTim, Siemens, Erlangen). The imaging protocol included:

1. High-resolution 3D T1-weighted MPRAGE scan with online motion correction (TR = 2,530 ms; TE = 2.5 ms; TI = 1,260 ms; FOV = 176 × 256 mm; voxel size = 1.0 × 1.0 × 1.0 mm)
2. Two runs of gradient echo EPI sequence fMRI (TR = 1.5 s, TE = 33 ms, FOV = 260 × 260, matrix = 104 × 104, voxel size = 2.5 × 2.5 × 2.5, slice thickness = 2.5, flip angle = 85°, multiband slice acceleration factor = 3, volumes = 240).

### Imaging Analyses

FSL Feat Version 6.03 was used for all fMRI analyses. Raw fMRI scans were preprocessed to correct for head motion (standard motion parameters using MCFLIRT; Jenkinson et al., 2002), spatially smoothed (4 mm extent threshold) and registered to the main structural image using boundary-based linear registration. The scans were also registered to standard MNI space using FNIRT nonlinear registration. Additionally, we used ICA-AROMA to further correct for motion-related artifacts (Pruim et al., 2015). We separated the task into two aspects: (1) prepare, when the participant listens to the word that they must subsequently repeat; and (2) speech, when the participant says the word out loud. Timing for each aspect was manually obtained for imaging analysis. Prepare timing was based off of the task timing, and speech timing was acquired by listening to the task audio recordings for each participant. We used multilevel general linear model analyses with mixed effects (FLAME 1) to identify regions of significant activation throughout the brain. First level analysis included the prepare and speech timing for each run at each time point.

Second-level analysis combined data from the two runs to compare speech-related functional activation between all MS participants to HC, as well as between the different MS subgroups. We will label each subgroup as follows:

**Table:** 

Healthy controls	HC
All multiple sclerosis participants	MS-all
MS control participants	MSC
MS participants with dysarthria	MS-dysarthria
MS participants with action tremor	MS-tremor
MS participants with dysarthria and tremor	MS-dystrem

This was completed for both prepare and speech timing to assess neural activation during speech preparation and motor speech production, respectively.

We used a z-stat threshold of >3.1 to identify clusters of significant activation (Bosnell et al., 2008; Eklund et al., 2016). This threshold was used to correct cluster size familywise error at *p* < 0.05.

### Statistical Analysis

Statistical analyses were performed using RStudio Version 4.2.2. Based on normality results, group comparisons on clinical and speech measures were made using the Kruskal-Wallis H test with Dunn’s test for multiple comparisons. Spearman correlations were then used to assess the connection between functional activation and measures of speech dysfunction and clinical presentation (EDSS, CBFSS and SARA scores). We used the 95th percentile of the z-scores for clusters with significant activation during speech production for each participant and correlated these with each clinical and speech metric.

## RESULTS

### Participant Demographics

Participant demographics for each group and subgroup are reported in Table 2. HC participants were age and sex matched to the MS participant group. EDSS scores differed significantly between MS-dystrem and MSC (H = 3.02, *p* = 0.01), MS-dysarthria (H = 3.38, *p* = 0.004) and MS-tremor (H = 3.15, *p* = 0.008). No other group differences were significant.

**Table 2.** Participant demographics including age, sex, disease severity (EDSS score), disease duration and disease course (progressive or relapsing-remitting)

	HC	MS-all	MSC	MS-dysarthria	MS-tremor	MS-dystrem
Sex (female %)	71.4	74.1	83.3	66.7	88.9	70.0
Age (mean, *SD*)	44.476 (14.504)	47.847 (11.177)	47.778 (9.927)	46.444 (15.685)	47.944 (11.963)	54.800 (6.374)
EDSS (median, IQR)	–	4.0 (2.5–6.0)	3.5 (2.0–5.5)	2.5 (1.8–3.8)	3.0 (2.0–5.04)	6.25 (5.42–6.54)
Disease duration (years, *SD*)	–	13.014 (7.732)	12.588 (8.239)	11.000 (6.708)	16.167 (10.700)	19.000 (8.641)
Disease course (progressive %)	–	59.2	61.1	33.3	58.8	60.0

*Note*. *SD* = standard deviation; EDSS = expanded disease severity scale; IQR = interquartile range.

### Comparing MS and Healthy Participants

MS participants performed worse on all clinical and speech measures when compared to HC (see Table 3). Only Vowel *f*0 CoV group differences were nonsignificant.

**Table 3.** Comparison of average performance on speech measures between MS and HC using the Kruskal-Wallis H test

	MS-all	HC	H	*p*
SARA	9.14 (7.95)	0.27 (0.73)	28.48	<0.001
SARA speech	0.67 (0.94)	0.00 (0.00)	8.247	0.004
Composite SARA	−10.7 (5.50)	−15.3 (3.49)	7.326	0.007
Composite EDSS	0.43 (0.92)	−0.56 (0.62)	11.79	0.001
DDK rate	5.56 (1.03)	6.22 (0.95)	4.660	0.031
Read rate	3.45 (0.63)	3.94 (0.32)	6.886	0.009
Vowel *f*0 CoV	0.95 (0.40)	0.91 (0.43)	0.311	0.577
Naturalness	0.64 (1.04)	0.00 (0.00)	6.243	0.012
Prolonged intervals	0.32 (0.64)	0.00 (0.00)	3.877	0.049

*Note*. Parentheses indicate standard deviation. SARA = Scale for the Assessment and Rating of Ataxia; EDSS = Expanded Disability Status Scale; DDK = diadochokinetic; *f*0 CoV = fundamental frequency coefficient of variation; MS-all = All multiple sclerosis participants; HC = healthy controls.

**Figure 2.**
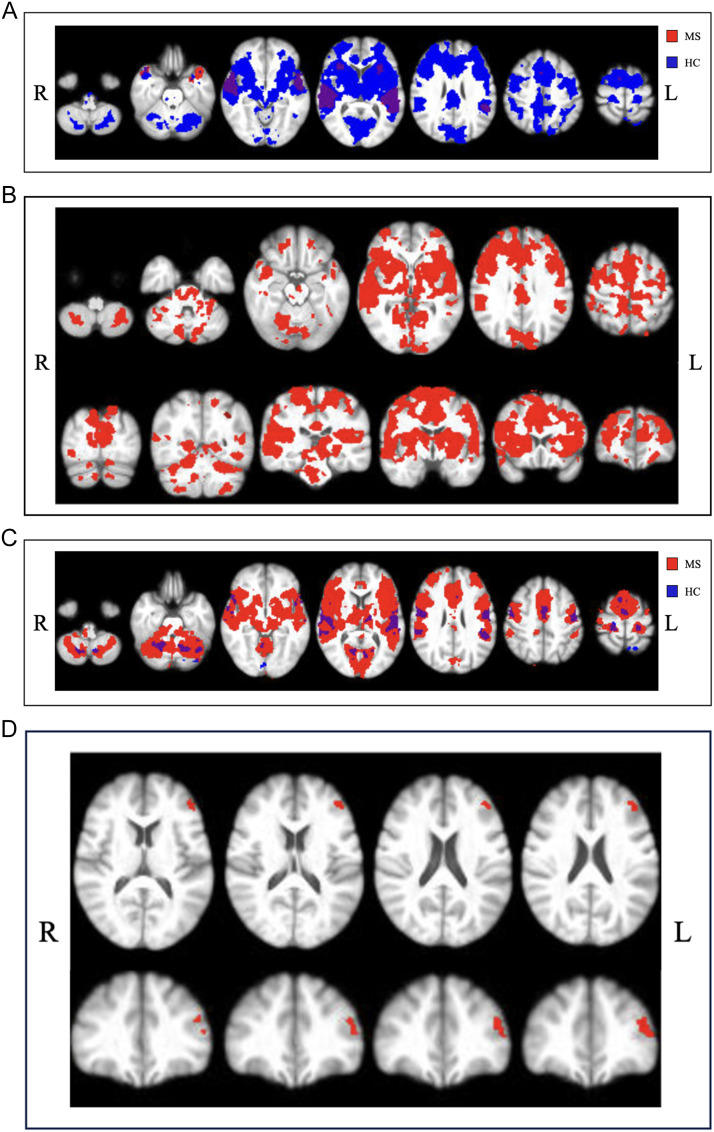
Activation during speech preparation. (A) Activation in people with multiple sclerosis (pwMS) in red and healthy controls (HC) in blue at z-threshold = 3.1 (*p* < 0.05). (B) Axial and coronal views of functional activation during speech preparation where MS-all < HC at z-threshold = 3.1 (*p* < 0.05). (C) Activation during speech production in pwMS and HC at z-threshold = 3.1 (*p* < 0.05). (D) Axial and coronal views of functional activation during speech production where MS-all > HC at z-threshold = 3.1 (*p* < 0.05).

People with MS displayed less extensive activation throughout the brain during speech preparation (activation during ‘prepare’, when asked to repeat the word) compared to HC (see Figure 2A, B). Conversely, during motor speech production (‘speech’), we found MS participants to have more extensive activation than HC. Functional activation during speech in pwMS was significantly different to HC (*z* = 3.94) in the left pars triangularis (BA45-L) and middle frontal gyrus (BA46-L; see Figure 2C, D). See Supplementary Tables S2 and S3 for peak activation cluster locations and statistics.

### Correlations With Measures of Clinical and Speech Dysfunction

We correlated functional activation in BA45/46-L during speech with activation in the same area during prepare, and with all clinical and speech metrics used to assess disease severity, cerebellar dysfunction and dysarthria in MS. In HC, activation of BA45/46-L during speech preparation correlated with activation during production (*ρ* = 0.622, *p* = 0.017). MS-all show this same correlation (*ρ* = 0.355, *p* = 0.031). Activation in BA45/46-L during speech production additionally correlated with SARA speech (*ρ* = 0.294, *p* = 0.028) and Naturalness (*ρ* = 0.436, *p* = 0.01) scores in the MS cohort. All other correlations between BA45/46-L activation during speech production and clinical and speech measures were found to be nonsignificant following corrections for multiple comparisons.

### Comparison Between MS Subgroups

Clinical and speech measures significantly differed between MS subgroups with the exception of vowel *f*0 CoV and composite EDSS (see Table 4). For all measures, MS-dystrem performed worse than other MS subgroups. Significance values provided refer to the overall significance of comparisons within each task. See Supplementary Table S2 for a detailed breakdown of all comparisons and their significance values.

**Table 4.** Average scores on clinical and speech scores and Kruskal-Wallis H scores based on MS subgroup

	MSC	MS-dysarthria	MS-tremor	MS-dystrem	H	*p*
SARA^a^^,^^b^	4.53 (3.66)	5.61 (4.63)	9.39 (5.90)	20.2 (8.92)	46.19	<0.001
CBFSS (median, IQR)^a^^,^^b^	1.5 (1.0–2.0)	1.0 (0.67–2.0)	2.0 (2.0–2.08)	2.5 (2.0–3.08)	17.46	<0.001
EDSS (median, IQR)^a^^,^^b^^,^^c^	3.5 (2.0–5.5)	2.5 (1.8–3.8)	3 (2.0–5.0)	6.25 (5.4–6.5)	14.33	<0.001
SARA speech^a^^,^^c^	0.44 (0.61)	0.56 (0.73)	0.28 (0.58)	1.9 (1.20)	25.91	<0.001
Composite SARA^a^	−13.7 (3.15)	−12.0 (3.25)	−11.1 (4.65)	−3.59 (6.4)	20.94	<0.001
Composite EDSS	0.08 (0.81)	0.43 (0.77)	0.32 (0.79)	1.35 (1.07)	18.54	0.239
DDK rate^a^^,^^b^	6.09 (0.75)	5.95 (0.71)	5.67 (0.71)	4.18 (0.99)	21.21	<0.001
Read rate^a^	3.81 (0.39)	3.57 (0.52)	3.44 (0.56)	2.80 (0.69)	18.42	0.001
Vowel f0 CoV	0.99 (0.39)	0.88 (0.36)	0.90 (0.46)	1.03 (0.32)	2.31	0.680
Naturalness^a^^,^^c^^,^^d^^,^^e^	0.00 (0.00)	1.11 (0.33)	0.00 (0.00)	2.40 (0.97)	63.97	<0.001
Prolonged intervals^a^^,^^b^^,^^c^	0.06 (0.25)	0.22 (0.44)	0.11 (0.32)	1.2 (0.92)	30.08	0.003

*Note*. Means and standard deviations are provided unless otherwise stated. SARA = Scale for the Assessment and Rating of Ataxia; CBFSS = Cerebellar and Brainstem Functional System Score; EDSS = Expanded Disability Status Scale; DDK = diadochokinetic; *f*0 CoV = fundamental frequency coefficient of variation.

^a^ Significant difference between MSC and MS-dystrem.

^b^ Significant difference between MS-dysarthria and MS-dystrem.

^c^ Significant difference between MS-tremor and MS-dystrem.

^d^ Significant difference between MS-dysarthria and MS-tremor.

^e^ Significant difference between MSC and MS-dysarthria.

**Figure 3.**
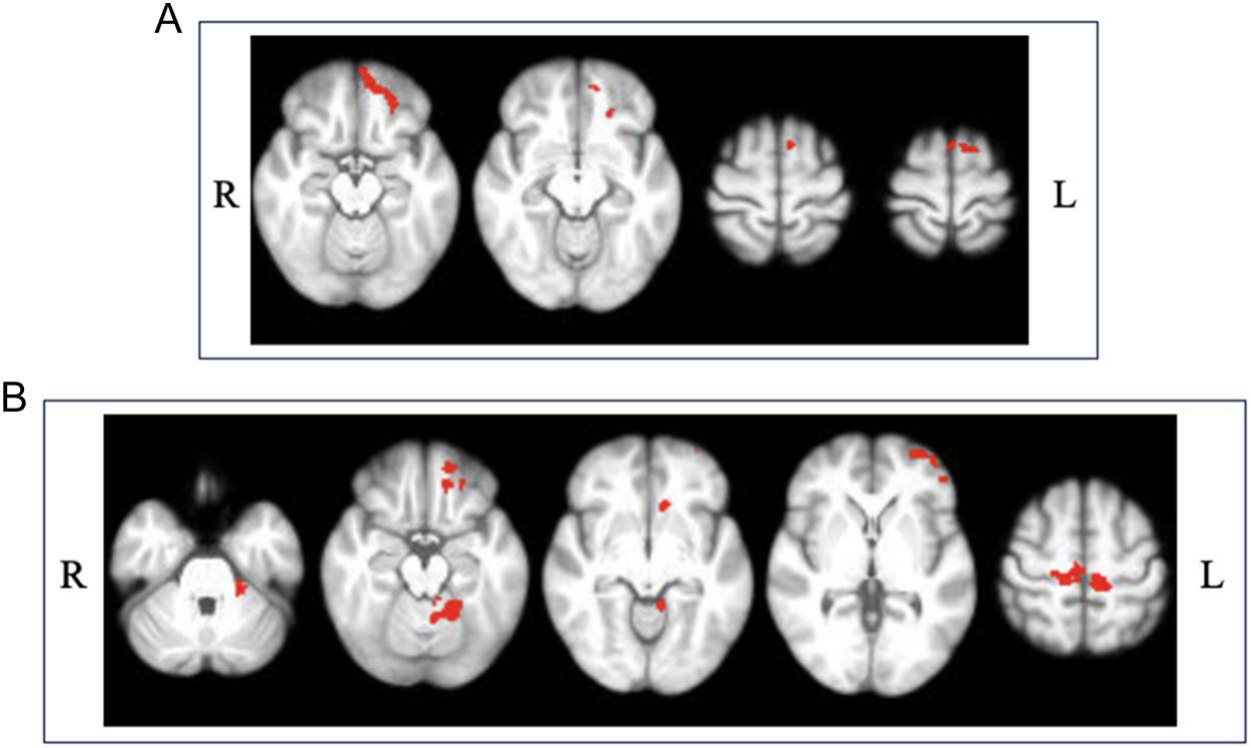
Functional activation during speech preparation. (A) Where MS-dysarthria > MSC at z-threshold = 3.1 (*p* < 0.05). (B) Where MS-dystrem > MSC at z-threshold = 3.1 (*p* < 0.05).

During speech preparation (prepare), MS participants with dysarthria (MS-dysarthria and MS-dystrem subgroups) displayed greater functional activity than MSC. Both dysarthria group participants had greater activation in the left frontal pole/orbitofrontal cortex, and left premotor cortex than those in the MS control group (see Figure 3A, B). Additionally, participants with both dysarthria and tremor showed greater functional activity in the bilateral primary motor and somatosensory cortices, left BA45 and left cerebellar lobules I–V (see Figure 3B). Peak coordinates and statistics can be found in Supplementary Table S3. No significant differences in functional activation were found between other MS subgroups.

**Figure 4.**
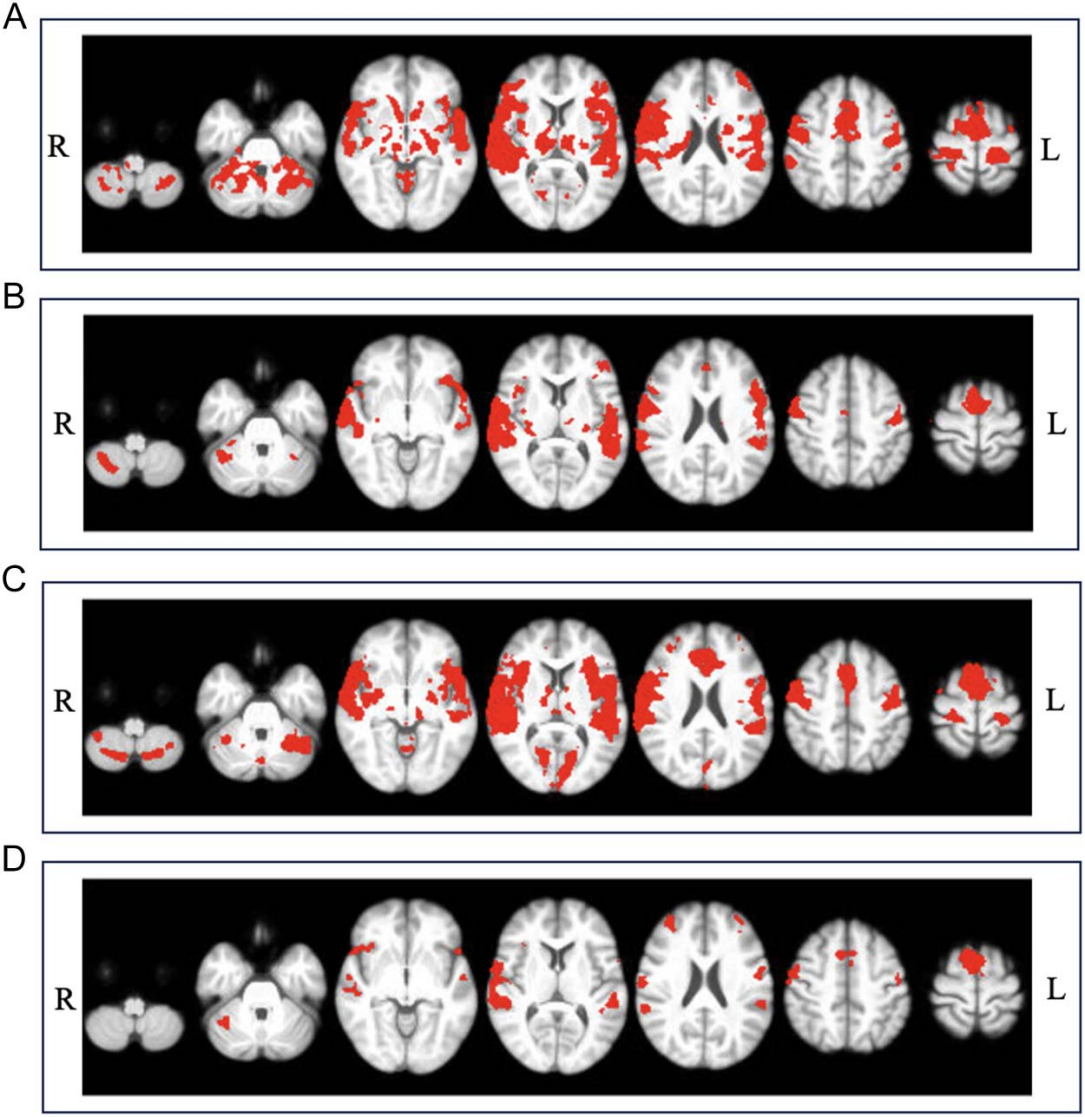
Functional activation during speech production. (A) In MS controls at z-threshold = 3.1 (*p* < 0.05). (B) In pwMS with clinical dysarthria at z-threshold = 3.1 (*p* < 0.05). (C) In pwMS with action tremor at z-threshold = 3.1 (*p* < 0.05). (D) In pwMS with dysarthria and tremor at z-threshold = 3.1 (*p* < 0.05).

**Figure 5.**
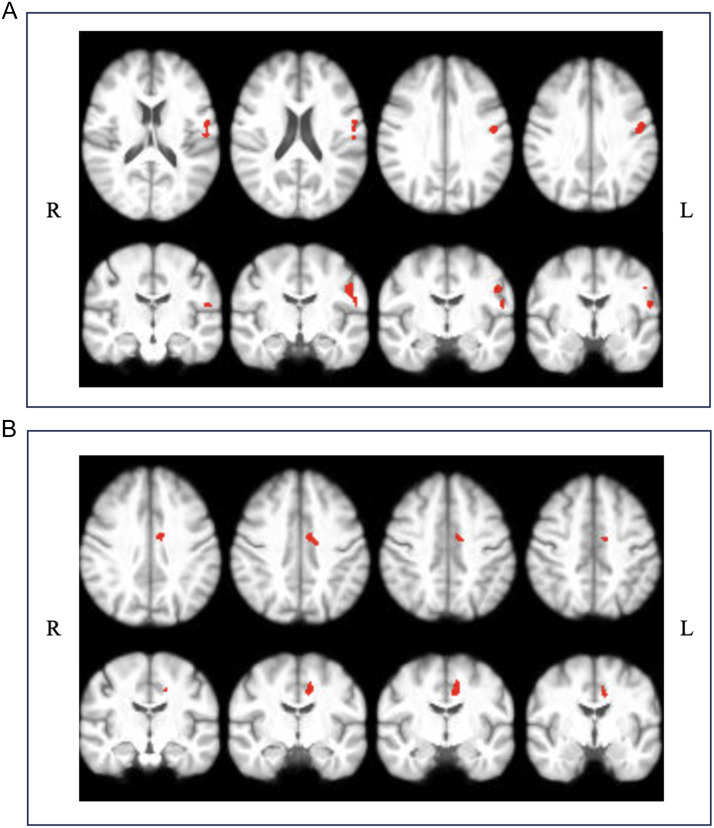
Axial and coronal views of functional activation during speech production. (A) Where MS-dystrem < MS-tremor at z-threshold = 3.1 (*p* < 0.05). (B) Where MS-dystrem < MSC at z-threshold = 3.1 (*p* < 0.05).

During speech production (speech), there were minimal differences in speech-related functional activation between the MS subgroups (see Figure 4A–D). We found that MS-tremor and MSC groups had greater functional activity during speech production than the combined MS-dystrem group (see Figure 5A, B). For MS-tremor, this was seen in the left primary somatosensory cortex (S1-L). For MSC, greater activity was located in the left SMA (SMA-L). See Supplementary Table S4 for peak activation cluster locations and statistics. No significant correlations between functional activity and either clinical or speech measures were identified.

## DISCUSSION

The present study aimed to determine the impact of MS on speech-related functional brain activation. To do this, we first compared speech production and brain activity during a word repetition task between pwMS and HC. We then further assessed the impact of cerebellar dysfunction on speech in MS, by assessing differences in clinical presentation, speech dysfunction and functional activation between subgroups of pwMS with dysarthria only, action tremor only, both dysarthria and action tremor, and neither dysarthria nor tremor.

### PwMS Have Altered Functional Activation During Speech Compared to HC

People with MS were found to have lower widespread functional activation during the preparatory stage of speech when compared to HC. We expected that the decreased functional activation during speech preparation in pwMS was likely due to anticipatory and motor neural network disruption. Higher functional activation has been seen in people with traumatic brain injury, where research has shown a difficulty in recruiting the anticipatory neural network (involving the dorsolateral prefrontal cortex, inferior parietal lobe and cerebellum; Ghajar & Ivry, 2008). Prior motor preparation research in MS also demonstrates delayed reaction time and sensorimotor and voluntary movement network disruption (Cabib et al., 2015; Leocani et al., 2005), further supporting our postulation that lower activity during speech preparation is due to motor network disruption. Similarly, a reduced readiness potential in areas including the cerebellum, basal ganglia and thalamus could contribute to this finding (Ghajar & Ivry, 2008; Gooijers et al., 2016). Changes in readiness potential have recently been documented in MS for the first time, where Bardel et al. (2022) found a correlation between readiness potential and both disease progression and motor performance in pwMS. Future research could examine readiness potential and its relationship to dysarthria in MS.

Compared to the activation differences observed during speech preparation, the opposite trend emerged during speech production. People with MS showed increased functional activation during active motor speech production compared to HC. The pattern of reduced preparation activation and increased execution activation during a motor task has been previously documented in people with moderate to severe traumatic brain injury (Gooijers et al., 2016). Higher activation during motor speech production was expected based on past motor fMRI research in an MS cohort (Boonstra, Noffs, et al., 2020; Lee et al., 2000; Rocca et al., 2002; Zeller & Classen, 2014). The significantly higher activity seen in pwMS was localized to the left BA45 and 46, both of which have been associated with aspects of speech production in previous research. A 2016 meta-analysis determined that BA45/46-L makes up part of a frontal language production system alongside the left BA44, BA47 and BA6 (the premotor cortex and SMA; Ardila et al., 2016). BA45-L has long been considered to play a key role in speech production (Broca, 1861; Flinker et al., 2015) and is thought to be involved in processing semantic information (Dapretto & Bookheimer, 1999; Goucha & Friederici, 2015), and syntactic processing alongside BA46-L (Ardila, 2012). BA46-L is thought to aid in executive control of speech production (Ardila et al., 2016). The findings, therefore, support our hypothesis that pwMS will have a higher level of functional activation during speech production than HC. The higher functional activity during speech production is likely a compensatory mechanism for damage, as increased functional activation is seen not only during motor tasks but also in cognitive assessment of pwMS (Audoin et al., 2003; Forn et al., 2007; López-Góngora et al., 2015; Mainero et al., 2006; Rocca & Filippi, 2007; Staffen et al., 2002) and dysarthria in other neurological disorders (Morgan et al., 2013; Rektorova et al., 2007). The increased activation in BA45/46-L was also correlated with worse performances on two speech measures, namely, the SARA speech and naturalness scores, both of which are indicative of clinical dysarthria in pwMS (Noffs et al., 2021).

Though there were no significant differences in cerebellar activation between pwMS and HC observed, pwMS scored higher on all measures indicative of cerebellar dysfunction and cerebellar speech dysfunction (SARA, SARA speech and composite SARA scores) compared to HC. A meta-analytic connectivity modeling study provided evidence toward language-related coactivation of the cerebellum and BA44 and BA45 in contralateral hemispheres (Bulut, 2022). Seed-voxel correlation patterns also highlight this connection between BA45-L and the right cerebellum (Tomasi & Volkow, 2012). While the studies mentioned were completed in healthy cohorts, the present study provides additional insight by demonstrating a relationship between cerebellar dysfunction (indicated by worse performance on SARA tasks) and altered functional activity within BA46/46-L during speech in pwMS.

### The Impact of Clinical Dysarthria and Action Tremor in MS

MS participants with naturalness scores indicative of dysarthria (MS-dysarthria and MS-dystrem) performed worse on assessments of speech dysfunction compared to pwMS without dysarthria (MSC and MS-tremor), mirroring previous findings on dysarthria in MS (Hartelius et al., 1995; Konstantopoulos & Karangioules, 2019; Noffs et al., 2018, 2021; Rusz et al., 2018). For those with action tremor as well as dysarthria (MS-dystrem), the dysfunction was more pronounced. The exceptions were with vowel frequency variability (vowel *f*0 CoV) and composite EDSS scores, where no significant group differences were identified. For clinical measures of MS, MS-dystrem again scored highest, demonstrating greater disease severity and cerebellar dysfunction than the other MS subgroups. As expected, MS-dystrem participants also had the highest disease duration of all MS participants (Boonstra et al., 2017; Wilkins, 2017).

Analysis of functional activation during the word repetition task showed few significant differences between MS subgroups. During speech preparation, MS participants with dysarthria (MS-dysarthria and MS-dystrem) had greater functional activation within the left frontal pole, orbitofrontal cortex and premotor cortex than MSC participants. MS-dystrem participants additionally had greater functional activation within the bilateral primary motor cortex, S1 and left anterior cerebellum compared to MSC. A 2019 study found that pwMS even without dysarthria may require greater planning for speech production (De Looze et al., 2019). Additionally, MS participants have higher readiness potential amplitudes than controls during a cognitive motor task (the Luria task; Bardel et al., 2022), indicating greater signal strength and longer movement preparation. This may be compensatory due to demyelination and accompanying neurodegeneration.

Conversely, MS-tremor and MSC participants had greater functional activation during speech production than MS-dystrem in S1-L and SMA-L, respectively. While these two regions are not generally thought of as primary speech or language areas, both are involved in the broader speech production network (Ardila et al., 2016; Behroozmand et al., 2015; Correia et al., 2020; Fuertinger et al., 2015; Guenther & Hickok, 2016). The S1 is involved specifically with somatosensory feedback control during speech production (Behroozmand et al., 2015; Guenther & Hickok, 2016), whereas the SMA appears to play a role in speech timing and initiation (Hertrich et al., 2016). Moreover, damage to the SMA can lead to word finding difficulties and inaccurate articulation and phonation (Hertrich et al., 2016). People with MS generally have greater activation than HC during motor tasks, but this activation is lower in pwMS with greater disease severity, reduced mobility, or over time as disease progresses (Ciccarelli et al., 2006; Pantano et al., 2005; Rocca & Filippi, 2007). More recent work on lower limb movement additionally shows lower functional activation of sensorimotor cortical regions in pwMS compared to controls (Strik et al., 2021). Given MS-dystrem participants have the greatest disease severity (EDSS), cerebellar dysfunction (CBFSS, SARA), longest disease duration, and worst speech performance of the MS subgroups, the lower functional activity during speech production in S1-L and SMA-L compared to other MS subgroups mirrors these findings. This may additionally explain the increase in functional activation seen in MS-dystrem and MS-dysarthria during speech preparation as compensatory due to hypoactivation during motor speech production.

### Limitations

Due to technical difficulties with the microphone within the MRI, some speech files were unintelligible, leading to a loss of data. Speech data from the fMRI word repetition task was lost fully for eight MS participants, with a further four missing data from one run. While we still had access to enough fMRI data for analysis, this data loss impacted the power of the study. Speech preparation functional activation data and speech and clinical data from these participants were still included. Further, due to the added noise from the MRI and microphone difficulties, we were unable to assess task performance for the fMRI speech task and could only obtain speech timing for analysis (whether the participant correctly paused for “listen” and articulated the correct word for “repeat”).

Most MS participants did not present with clinical dysarthria, with nine participants in the MS-dysarthria and 10 in the MS-dystrem subgroups for analysis. A majority of these participants had only mild levels of dysarthria. The lack of variation in level of speech dysfunction likely impacted the comparisons between MS subgroups. We therefore expect the minimal differences seen in functional activation between MS subgroups are due to low power and suggest further research with a larger cohort. Similarly, the greater extent of functional activation in HC during speech preparation may be due to the smaller sample size in comparison to MS-all, meaning the control group had greater natural variation. Moreover, while possible with the current data, running regression analyses between behavioral factors and functional activation within a larger and more diverse MS cohort (with more varied disease duration and severity) would elucidate and expand on these findings.

The lack of a specific cognitive assessment during this study should also be considered. Previous research into speech and cognition in MS has shown a relationship between articulation speed and mean length of utterances, and cognitive function, particularly information processing speed (Arnett et al., 2008; Feenaughty, 2023; Feenaughty et al., 2013). Feenaughty et al. (2021) found that pwMS with dysarthria and mild cognitive impairment have slower speech rate than pwMS with dysarthria and no cognitive impairment, thus illustrating the impact of cognitive difficulties on speech production. Given we did not control for any cognitive aspect outside of separating speech preparation and production, we cannot say our findings reflect purely motor processes. We suggest that further research using fMRI to assess dysarthria in MS ought to either control for cognitive impairment or include cognition as a variable of interest.

### Conclusion

Our research offers insight into the impact of MS on speech and speech-related functional activity. We found that pwMS require greater activation of BA45/46-L, key regions for speech processing and control, than HC during word repetition. This activity indicates a compensatory mechanism due to MS-related damage, lower functional activity during speech preparation, or both. Increased BA45/46-L activity in pwMS also correlated with measures of dysarthria. We additionally found that pwMS with greater cerebellar involvement—indicated by presenting with both dysarthria and tremor—had lower S1-L and SMA-L activation during speech than other pwMS. We expect this is due to the greater level of disease severity in these participants. To further assess the impact of cerebellar dysfunction on speech-related functional activation in pwMS, we suggest future research ought to focus on a larger cohort of pwMS with varying levels of clinical cerebellar dysarthria.

## FUNDING INFORMATION

Anneke van der Walt, National Health and Medical Research Council (AU), Award ID: 1085461.

## AUTHOR CONTRIBUTIONS

**Katherine Hope Kenyon**: Formal analysis: Lead; Investigation: Lead; Methodology: Equal; Validation: Lead; Visualization: Lead; Writing – original draft: Lead; Writing – review & editing: Lead. **Frederique Boonstra**: Data curation: Lead; Methodology: Equal; Project administration: Equal; Supervision: Supporting; Writing – review & editing: Supporting. **Gustavo Noffs**: Formal analysis: Equal; Supervision: Supporting; Writing – review & editing: Supporting. **Angela Morgan**: Methodology: Equal; Writing – review & editing: Supporting. **Adam Vogel**: Conceptualization: Equal; Supervision: Supporting; Writing – review & editing: Supporting. **Scott Kolbe**: Conceptualization: Equal; Formal analysis: Supporting; Methodology: Equal; Supervision: Supporting; Writing – review & editing: Supporting. **Anneke van der Walt**: Conceptualization: Lead; Funding acquisition: Lead; Methodology: Equal; Supervision: Lead; Writing – review & editing: Supporting.

## CONFLICT OF INTEREST STATEMENT

Anneke van der Walt served on advisory boards and receives unrestricted research grants from Novartis, Biogen, Merck and Roche. She has received speaker’s honoraria and travel support from Novartis, Roche and Merck. She receives grant support from the National Health and Medical Research Council of Australia and MS Research Australia. Adam Vogel is Chief Science Officer of Redenlab, Inc. Gustavo Noffs works in scientific development for Redenlab, Inc. Scott Kolbe received unrestricted research grants from Biogen and grant support from MS Research Australia. Other authors declare that they have no known competing financial interests or personal relationships that could have appeared to influence the work reported in this paper.

## DATA AVAILABILITY STATEMENT

Anonymized data available at https://github.com/kkneuro/speech-fMRI.

## Supplementary Material



## TECHNICAL TERMS

Multiple sclerosis: A neurodegenerative disorder in which the immune system attacks myelin, leading to deficits in cognition and motor control.
Demyelination: Damage to the neuron’s insulating layer—the myelin sheath—which disrupts the conduction of electrical signals within the central nervous system.
Dysarthria: A speech disorder caused by reduced strength and control of the muscles involved in articulation and breathing.
Functional magnetic resonance imaging (fMRI): An imaging technique that visualizes brain activity through measuring changes in brain metabolism over time, usually changes in blood oxygen levels.
Neurodegeneration: Progressive loss of either the structure or function of neurons, which leads to changes in and loss of brain function.
