# Supplementary material for: Altered Functional Activation Is Associated With Speech Dysfunction in People With Multiple Sclerosis
Source: Neurobiol Lang (Camb). 2025 Nov 13;6:NOL.a.23. doi: 10.1162/NOL.a.23 (PMC12618014; doi:10.1162/NOL.a.23)
Supplement: Supplementary file 1 [file nol-6-1-23-s001.pdf]

Supplementary table 1: Word list for motor speech task

**ITEM**	**R/L**	**WORD**
1	Repeat	Calendar
2	Listen	Yesterday
3	Listen	Cab
4	Repeat	Butter
5	Listen	Magic
6	Repeat	Honey
7	Repeat	Negative
8	Listen	People
9	Repeat	Fantastic
10	Repeat	Neck
11	Repeat	Tail
12	Listen	Charity
13	Repeat	People
14	Repeat	Justify

15	Repeat	Mess
16	Listen	Tail
17	Listen	Neck
18	Listen	Fantastic
19	Repeat	Charity
20	Repeat	Magic
21	Repeat	Cab
22	Listen	Mess
23	Repeat	Author
24	Listen	Negative
25	Listen	Butter
26	Listen	Honey
27	Listen	Justify
28	Repeat	Yesterday
29	Listen	Author

30	Listen	Calendar
31	Listen	Food
32	Repeat	Logical
33	Listen	Coin
34	Listen	Threatening
35	Repeat	Crash
36	Listen	Crash
37	Listen	Broken
38	Repeat	Kitchen
39	Repeat	Zero
40	Listen	Hospital
41	Repeat	Fruit
42	Listen	Fruit
43	Listen	Glory
44	Repeat	Dish

45	Repeat	Finger
46	Repeat	Threatening
47	Listen	Kitchen
48	Repeat	Visitor
49	Repeat	Hospital
50	Repeat	Food
51	Listen	Visitor
52	Listen	Logical
53	Listen	Dish
54	Repeat	Broken
55	Listen	Gab
56	Repeat	Glory
57	Repeat	Coin
58	Repeat	Gab
59	Listen	Finger
60	Listen	Zero

Supplementary table 2: Average scores on clinical and speech measures, and Kruskal-Wallis H scores based on MS subgroup.

Test	Chi^2	df	kw-p	dunn_group1	dunn_group2	group1_n	group2_n	p.adj
SARA	46.191	4	2.247E-09	HC	MS_control	13	18	0.0163
				HC	MS_dys	13	9	0.0215
				HC	MS_tremor	13	18	0.00000336
				HC	MS_dystremor	13	10	3.32E-09
				MS_control	MS_dys	18	9	0.72
				MS_control	MS_tremor	18	18	0.0916
				MS_control	MS_dystremor	18	10	0.00067
				MS_dys	MS_tremor	9	18	0.268
				MS_dys	MS_dystremor	9	10	0.0156
				MS_tremor	MS_dystremor	18	10	0.133
CBFSS	17.46	3	0.0005682	MS_control	MS_dys	16	9	0.685
				MS_control	MS_tremor	16	18	0.0916
				MS_control	MS_dystremor	16	10	0.0029
				MS_dys	MS_tremor	9	18	0.0916
				MS_dys	MS_dystremor	9	10	0.00303
				MS_tremor	MS_dystremor	18	10	0.202
EDSS	14.328	3	0.002491	MS_control	MS_dys	18	9	1
				MS_control	MS_tremor	18	18	1
				MS_control	MS_dystremor	18	10	0.0102
				MS_dys	MS_tremor	9	18	1
				MS_dys	MS_dystremor	9	10	0.00437
				MS_tremor	MS_dystremor	18	10	0.00805
SARA speech	25.912	4	0.00003296	HC	MS_control	13	18	0.288

				HC	MS_dys	13	9	0.288
				HC	MS_tremor	13	18	0.967
				HC	MS_dystremor	13	10	0.0000121
				MS_control	MS_dys	18	9	0.967
				MS_control	MS_tremor	18	18	0.967
				MS_control	MS_dystremor	18	10	0.00643
				MS_dys	MS_tremor	9	18	0.967
				MS_dys	MS_dystremor	9	10	0.0675
				MS_tremor	MS_dystremor	18	10	0.000379
Composite SARA	20.94	4	0.0003255	HC	MS_control	10	14	1
				HC	MS_dys	10	7	0.498
				HC	MS_tremor	10	18	0.119
				HC	MS_dystremor	10	8	0.000206
				MS_control	MS_dys	14	7	1
				MS_control	MS_tremor	14	18	0.499
				MS_control	MS_dystremor	14	8	0.0022
				MS_dys	MS_tremor	7	18	1
				MS_dys	MS_dystremor	7	8	0.119
				MS_tremor	MS_dystremor	18	8	0.0891
Composite EDSS	18.536	4	0.0009695	HC	MS_control	11	15	0.221
				HC	MS_dys	11	9	0.0861
				HC	MS_tremor	11	18	0.051
				HC	MS_dystremor	11	8	0.000299
				MS_control	MS_dys	15	9	1
				MS_control	MS_tremor	15	18	1
				MS_control	MS_dystremor	15	8	0.0861
				MS_dys	MS_tremor	9	18	1
				MS_dys	MS_dystremor	9	8	0.41

				MS_tremor	MS_dystremor	18	8	0.221
DDK rate	21.211	4	0.0002876	HC	MS_control	12	17	1
				HC	MS_dys	12	7	1
				HC	MS_tremor	12	18	0.342
				HC	MS_dystremor	12	10	0.000397
				MS_control	MS_dys	17	7	1
				MS_control	MS_tremor	17	18	0.636
				MS_control	MS_dystremor	17	10	0.000768
				MS_dys	MS_tremor	7	18	1
				MS_dys	MS_dystremor	7	10	0.0294
				MS_tremor	MS_dystremor	18	10	0.0543
Read rate	18.42	4	0.001021	HC	MS_control	13	15	1
				HC	MS_dys	13	9	0.486
				HC	MS_tremor	13	18	0.132
				HC	MS_dystremor	13	10	0.000917
				MS_control	MS_dys	15	9	1
				MS_control	MS_tremor	15	18	0.433
				MS_control	MS_dystremor	15	10	0.00702
				MS_dys	MS_tremor	9	18	1
				MS_dys	MS_dystremor	9	10	0.238
				MS_tremor	MS_dystremor	18	10	0.301
Naturalness	63.972	4	4.236E-13	HC	MS_control	13	16	1
				HC	MS_dys	13	9	0.0000509
				HC	MS_tremor	13	18	1
				HC	MS_dystremor	13	10	1.29E-07
				MS_control	MS_dys	16	9	0.0000262
				MS_control	MS_tremor	16	18	1
				MS_control	MS_dystremor	16	10	3.38E-08

				MS_dys	MS_tremor	9	18	0.0000193
				MS_dys	MS_dystremor	9	10	1
				MS_tremor	MS_dystremor	18	10	1.7E-08
Prolonged intervals	30.077	4	0.000004721	HC	MS_control	13	16	1
				HC	MS_dys	13	9	1
				HC	MS_tremor	13	18	1
				HC	MS_dystremor	13	10	0.0000118
				MS_control	MS_dys	16	9	1
				MS_control	MS_tremor	16	18	1
				MS_control	MS_dystremor	16	10	0.0000231
				MS_dys	MS_tremor	9	18	1
				MS_dys	MS_dystremor	9	10	0.00673
				MS_tremor	MS_dystremor	18	10	0.0000508

Supplementary table 3: Peak cluster locations and statistics for functional activation during "prepare"

Contrast	Region	Size (vx)	Z	Peak voxel location			Peak NMI coordinates		
				x	y	z	x	y	z
dystrem > MSC	L pre- and postcentral gyri	120	4.24	51	47	63	-18	-44	-20
	R pre- and postcentral gyri	141	4.25	39	80	64	-26	38	-16
	L frontal pole, IFG (BA45)	145	4.36	60	92	36	-30	58	0
	L frontal pole, OFC	173	4.31	58	82	28	-26	38	-16
	L cerebellar lobules I-V	331	4.96	54	41	26	-18	-44	-20
dysarthria > MSC	L premotor cortex	100	4.32	49	67	73	-8	8	74
	L frontal pole, OFC	158	4.62	47	93	27	-4	60	-18

MSC: MS controls; dystrem: MS participants with dysarthria and tremor; L: left hemisphere; R: right hemisphere; IFG: inferior frontal gyrus; BA45: Brodmann area 45; OFC: orbitofrontal cortex.

Supplementary table 4: Peak cluster locations and statistics for functional activation during "speech"

Contrast	Region	Size (vx)	Z	Peak voxel location			Peak NMI coordinates		
				x	y	z	x	y	z
MS > HC	L IFG (BA45/46)	84	4.02	69	83	42	-44.5	38.9	17.2
dystrem < MS-tremor	L primary somatosensory cortex	151	3.99	73	58	46	-56	-10	20
dystrem < MSC	L supplementary motor	81	4.11	50	60	55	-10.2	-7.95	40.1

MS-all: all MS participants; HC: healthy controls; MSC: MS controls; MS-tremor: MS participants with tremor; dystrem: MS participants with dysarthria and tremor; L: left hemisphere; R: right hemisphere; IFG: inferior frontal gyrus; BA45/46: Brodmann areas 45 and 46
